# Comparison of outcomes at 6 weeks following postpartum intrauterine contraceptive device insertions by doctors and nurses in India: a case–control study^☆^^☆☆^

**DOI:** 10.1016/j.contraception.2015.12.012

**Published:** 2016-04

**Authors:** Vivek Yadav, Sudharsanam Balasubramaniam, Saswati Das, Ashish Srivastava, Ashish Srivastava, Somesh Kumar, Bulbul Sood

**Affiliations:** a Jhpiego — an affiliate of Johns Hopkins University, 221, Okhla Phase III, New Delhi 110020, India; b Jhpiego — an affiliate of Johns Hopkins University, 270, 1st Floor, Patliputra Colony, Patna, Bihar 800013, India

**Keywords:** Task sharing, Postpartum family planning, Postpartum intrauterine contraceptive device, Nurses, Midwives

## Abstract

**Objective:**

As part of a strategy to revitalize postpartum family planning services, Government of India revised its policy in 2013 to permit trained nurses and midwives to insert postpartum intrauterine contraceptive devices (PPIUCDs). This study compares two key outcomes of PPIUCD insertions — expulsion and infection — for physicians and nurses/midwives to generate evidence for task sharing.

**Study design:**

We analyzed secondary data from the PPIUCD program in seven states using a case–control study design. We included facilities where both doctors and nurses/midwives performed PPIUCD insertions and where five or more cases of expulsion and/or infection were reported during the study period (January–December 2013). For each case of expulsion and infection, we identified a time-matched control who received a PPIUCD at the same facility and had no complaints. We performed a multiple logistic regression analysis focusing on provider cadre while controlling for potential confounding factors.

**Results:**

In 137 facilities, 792 expulsion and 382 infection cases were matched with 1041 controls. Provider type was not significantly associated with either expulsion [odds ratio (OR) 1.84; 95% confidence interval (CI): 0.82–4.12] or infection (OR 0.73; 95% CI: 0.39–1.37). Compared with centralized training, odds of expulsion were higher for onsite (OR 2.32, 95% CI: 1.86–2.89) and on-the-job training (OR 1.23, 95% CI: 1.11–1.36), but odds of infection were lower for onsite (OR 0.45, 95% CI: 0.27–0.75) and on-the-job training (OR 0.31, 95% CI: 0.25–0.37).

**Conclusion:**

Trained nurses and midwives who conduct deliveries at public health facilities can perform PPIUCD insertions as safely as physicians.

**Implications:**

Institutional deliveries are increasing in India, but most normal vaginal deliveries at public health facilities are attended by nurses and midwives due to a shortage of physicians. Task sharing with nurses and midwives can increase women's access to and the acceptability of quality PPIUCD services.

## 1 Introduction

Using family planning (FP) to space births at least 36 months apart can avert 30% of maternal deaths and 10% of child deaths [1], [2]. In India, however, only 26% of postpartum women are using contraceptives [3] and more than 60% of births follow an interval of less than 36 months [4]. Sixty-five percent of postpartum women in India have an unmet need for contraception to delay or limit future pregnancies [3].This is similar to the levels of unmet need across 27 countries [5]. Sterilization has remained the leading method of contraception in India, accounting for 40% of FP users [6], [4], but it does not address women's needs for healthy birth spacing. The postpartum intrauterine contraceptive device (PPIUCD) — a long-acting, reversible contraceptive — offers a safe, effective and convenient alternative [7]. It has also been found to be acceptable among Indian women [8], [9].

In the last decade, more and more women chose to give birth in health institutions. Proportion of deliveries taking place at health facilities increased from 41% in 2005–2006 [4] to 86.9% [10]. This preference has emerged due to the government's flagship program — Janani Suraksha Yojana, a conditional cash transfer scheme for promoting institutional deliveries. It is a part of government's efforts to reduce maternal and neonatal mortality under the National Health Mission [11].

Given high unmet need for birth spacing and the rise in institutional deliveries, the Government of India, with technical support from Jhpiego, has been working to reinvigorate and scale up the use of postpartum FP, with a focused effort on expanding the capacity to provide PPIUCD services. Appropriate provision of postpartum FP services includes antenatal counseling, peripartum support for initiating a method and postpartum guidance to successfully continue use. Institutional deliveries create a unique opportunity to offer a long-acting yet reversible method of contraception to women immediately following their childbirth. Delaying insertions until later is less effective because most clients tend not to return to facilities for FP services [12]. Cost is not a barrier for women because FP services in India, including PPIUCDs, are provided free of charge at government health facilities. Limited availability of skilled human resources — which are essential to ensure the quality of PPIUCD services — poses a challenge for increasing access to this safe and highly effective method. A 2012 Bottleneck Analysis identified the shortage of skilled providers as a key barrier to implementing effective interventions for improved maternal and newborn health in India [13]. Task sharing, which is a globally accepted solution for accelerating access to health services, was identified as a viable strategy to expand the provider base and make postpartum FP services available to all women delivering at health facilities. Task sharing refers to *giving additional training to existing cadres of providers and then allowing them to take activities they have not undertaken before* [14]. Nurses and midwives (auxiliary nurse midwife and general nurse midwife) attend the majority of normal vaginal births at health facilities in India [15], but provision of PPIUCD services was initially limited to doctors. Evidence from several countries supports task sharing in the delivery of FP services [16], [17], and studies have found that provision of interval intrauterine contraceptive devices (IUCDs) by nurse-midwives is effective and feasible in low-resource settings [18], [19], [20]. World Health Organization has also recommended the option of insertion of IUCDs by nurses and midwives [14].

In order to rapidly scale up PPIUCD services in India, the government changed policy in 2013 to allow trained nurses and midwives to insert PPIUCDs [21] and initiated capacity building of nursing staff to provide PPIUCD services. Enhanced focus on PPIUCD has increased the uptake of PPIUCD services; with 120,000 insertions in 2012–2013 to approximately 300,000 insertions in 2013–2014 [22]. The objective of this paper is to generate country-level evidence on the safety and effectiveness of task sharing for PPIUCD insertions based on this experience. Our analysis compares the outcomes of insertions performed by physicians with those performed by nurses and midwives.

## 2 Materials and methods

This study is a retrospective analysis of secondary data on two outcomes of PPIUCD insertion — expulsion and infection using a case–control study design. The data were collected as part of routine monitoring of postpartum FP programs implemented by the Government of India, with technical support from Jhpiego, in high priority states that lag behind on key health indicators. These seven high-focus states are Bihar, Uttar Pradesh, Madhya Pradesh, Rajasthan, Chhattisgarh, Uttarakhand and Jharkhand. The program includes training doctors, nurses and midwives who provide maternity services at public health facilities with a purpose to expand access to PPIUCD. The maternity service providers are trained using a 3-day standardized curriculum that, along with theoretical knowledge, focuses on insertion competency: first on an anatomical model and then graduating to an actual client. In the program, three training approaches are deployed for provider trainings: (a) centralized, (b) onsite and (c) on-the-job. In centralized trainings, select providers undergo training led by recognized trainers at established state or divisional training sites — which are usually tertiary-level care facilities with high caseloads. Onsite trainings are conducted at the actual workplace of the providers by Jhpiego training team with support from existing trained providers, recognized as champions, who have successfully implemented the program at their respective facility after centralized trainings. On-the-job approach refers to an informal, peer-to-peer instruction, in which a trained PPIUCD provider trains colleagues and subordinates in a particular facility at their own determined pace but ensuring competency of skills as per the standardized checklist.

Service data come from public health facilities, including primary health centers, community health centers, district and subdistrict hospitals and medical colleges. We included facilities in the study if (1) both doctors and nurses (or midwives) performed PPIUCD insertions and (2) the facility reported at least five cases of expulsion and/or infection in the 6 weeks following PPIUCD insertions during the calendar year 2013. We chose five as the threshold as it was the median number of expulsions and/or infections reported at 6 weeks follow-up from facilities providing PPIUCD services in 2013.

Of the 366 facilities where Jhpiego was working in 2013, 137 facilities (38%) met these criteria and were included in the study.

**Fig. 1:**
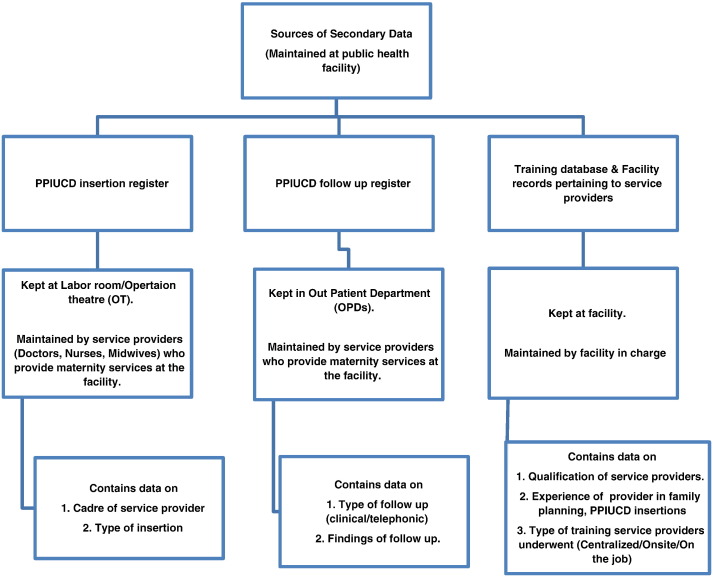
Figure describing the sources of secondary data utilized for the study.

We took data from facility registers exclusively devoted to PPIUCD insertion and follow-up services, a training database and other facility records pertaining to service providers. The PPIUCD insertion and follow-up registers are maintained by the maternity service providers of the facility whereas the training database and other facility records are maintained by the office of the facility in-charge. We trained and periodically mentored all maternity service providers and other data handlers to maintain the quality of service data during the rollout of PPIUCD services at the facilities included in this study We collected information on the cadre of provider who inserted the PPIUCD and the type of insertion (postplacental, postpartum or intracesarean) from PPIUCD insertion registers. Postplacental insertion refers to insertion performed within 10 min of expulsion of placenta following a vaginal delivery whereas postpartum insertion is done after 10 min but within 48 h of delivery. We collected information on type of follow-up (clinical visit or telephone) and findings of the follow-up (continuation/expulsion/infection) from the PPIUCD follow-up registers. Training database and facility records provided information on qualification of service providers, experience of service providers in FP and PPIUCD services and the type of training service providers underwent (Fig. 1). We found that information on providers' years of experience in offering FP and PPIUCD services are incomplete at many facilities.

**Fig. 2:**
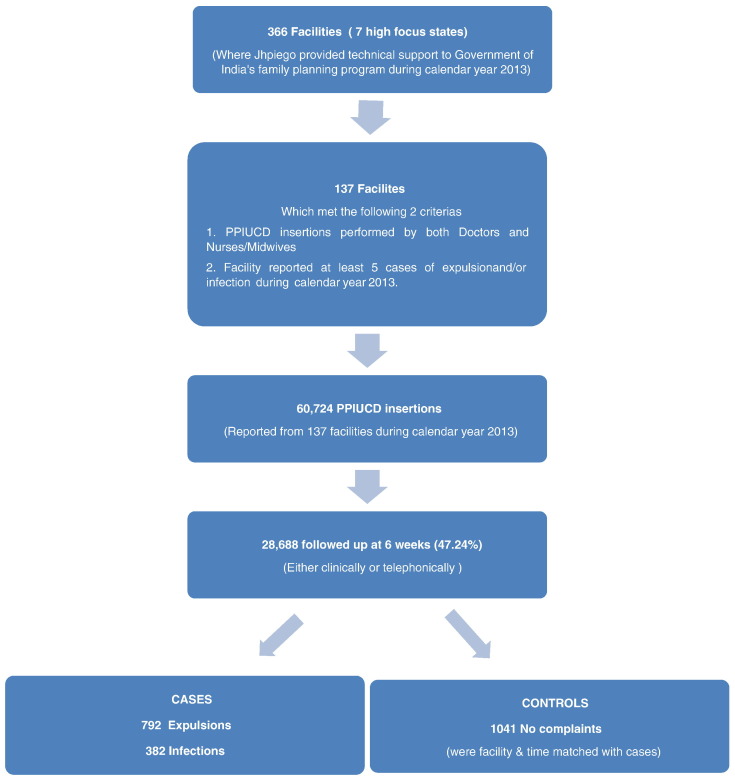
Flowchart depicting selection of cases and controls.

The PPIUCD insertion registers for 2013 in the selected 137 facilities recorded 60,724 PPIUCD insertions. Of these, 6-week follow-up data of 28,688 clients were recorded in the PPIUCD follow-up registers. Out of these 28,688 follow-ups, we included all cases of expulsion and infection between January and December 2013 in the dataset for analysis; we selected these outcomes because they are directly related to the skills and practices of the providers [7], [23]. For each case of expulsion and infection, we identified a control who (1) received a PPIUCD at the same facility either during the 2 months preceding or 2 months following the month of the case's insertion and (2) had no complaints at 6-week follow-up (Fig. 2). We listed all possible controls for cases during a month and if more than one control was available per case, we selected the controls by systematic sampling. We divided the number of cases by the number of possible controls to obtain a number “*n*” (rounded off to nearest and smallest whole number). From the list of controls, we randomly chose the first control such that its serial number was less than “*n*”. Subsequently, every *n*th control was chosen from the list. We included a total of 792 cases of expulsion, 382 cases of infection and 1041 controls in the final analysis after cleaning of the dataset. Cleaning of dataset involved removing those cases and controls that did not have the information on cadre of provider who inserted the PPIUCD. All women received the Copper T 380A.

Among cases and controls that were included in the final analysis, most women (73.7%) had returned to the facility for a follow-up visit after 6 weeks of receiving the PPIUCD. The follow-up for the remaining women (26.2%) was telephonic. During clinic or facility follow-up, service providers diagnosed infection or expulsion using a standard protocol, whereas in case of telephonic follow-up, service providers made the diagnosis based on self-report of the clients using a standard protocol.

Our research team visited each facility and identified cases and controls from PPIUCD registers. They ensured the quality of the data from PPIUCD follow-up registers by cross-verification with PPIUCD insertion registers. We linked client level data to information from other databases and deidentified it. We entered data in Microsoft Excel and then analyzed using Stata 13.0 (StataCorp. 2013. *Stata Statistical Software: Release 13*. College Station, TX: StataCorp LP).

Our analysis generated proportions for categorical variables and cases and controls were compared using a chi-square test. We computed mean and standard deviations for continuous variables, and we compared cases and controls using an independent sample *t* test. We calculated unadjusted odds ratios (ORs) and 95% confidence intervals (CIs) using simple logistic regression, accounting for clustering at a state level. We adjusted for clustering at state level as number of observations varied across states. The study design required one control for each case. After compiling and cleaning of data, we had more than one control for each outcome (expulsion and infection). Hence, we used all controls for both outcomes without making any further selection. We included the primary outcome of interest, provider cadre, in a multiple logistic regression model along with following potential confounding factors — type of insertion, type of training, type of facility and type of follow-up. We put all these potential confounding factors in the final regression model irrespective of the results of univariate analysis. We calculated adjusted ORs with 95% CIs (using robust standard errors). We classified all providers with a qualification in midwifery or nursing as nurses and those medical graduates and postgraduates as doctors. The maximum allowable alpha error was set as 5%. We could not adjust for clustering by provider in the model because the data sources did not include a provider identification number or standardized provider name. However, we matched cases and controls by facility, and we adjusted for clustering at the state level.

We obtained the approval for secondary data analysis from the institutional review board of the Johns Hopkins Bloomberg School of Public Health.

## 3 Results

A total of 60,724 PPIUCDs were inserted at the 137 facilities in the study; they yielded 792 cases of expulsion and 382 cases of infection. These cases were matched with 1041 controls who had no complaints at 6-week follow-up after PPIUCD insertion.

The mean age of expulsion cases (24.2 years, SD 3.3 years) and controls (24.4 years, SD 3.6 years) was similar. However, infection cases (24.9 years, SD 3.8) were significantly older than controls (24.4 years, SD 3.6 years) (p = .02). Nurses performed 59.3% of all 2215 insertions included in the analysis.

Table 1 shows the distribution of independent variables that may be related to PPIUCD outcomes among expulsion cases and controls. Most varied significantly between case and control groups, including state, facility type, provider qualification, provider's years of experience offering FP services, type of PPIUCD training, type of insertion and type of follow-up. Only one variable — provider's years of experience offering PPIUCD insertions — did not vary significantly between expulsion cases and controls.

Table 2 shows the distribution of infection cases and controls by independent variables that may be related to PPIUCD outcomes. Five variables — state, provider qualification, provider's experience offering FP services, type of training and type of follow-up — varied significantly between the case and control groups. There was no statistically significant difference by facility type, provider's experience with PPIUCD insertions and type of insertion.

Table 3 presents the results of the logistic regression analyses for expulsion. In the simple logistic regression, the difference in expulsion risks following PPIUCD insertion by nurses and doctors was statistically significant (OR 2.06, 95% CI: 1.69–2.50). On accounting for clustering by state, the difference became nonsignificant (OR 2.06, 95% CI: 0.99–4.26). After adjustment for confounders like type of insertion, type of training, type of facility and the type of follow-up in the multiple regression analysis, cadre remained nonsignificant (OR 1.84, 95% CI: 0.82–4.12). We also did a multiple regression analysis for expulsion with 1:1 matching of cases and controls (792 expulsion cases with 792 facility- and time-matched controls) in which the cadre remained nonsignificant (OR 0.96, 95% CI: 0.71–1.30). The odds of expulsion were significantly higher for postplacental insertion (OR 1.86, 95% CI: 1.01–3.43) and postpartum insertion (OR 2.16, 95% CI: 1.12–4.17) compared with intracesarian insertion in the simple regression, but this variable did not remain significant after adjusting for confounders. In contrast, type of PPIUCD training remained statistically significant even after adjusting for confounders; the odds of expulsion were higher for both onsite training (OR 2.32, 95% CI: 1.86–2.89) and on-the-job training (OR 1.23, 95% CI: 1.11–1.36) compared with centralized training in the multiple regression.

Table 4 presents the results of the logistic regression analyses for infection following PPIUCD insertion. There was no statistically significant association between cadre and infection in the simple logistic regression (OR 0.91 for nurses and midwives, 95% CI: 0.73–1.16), simple logistic regression after accounting for clustering by state (OR 0.91 for nurses and midwives, CI: 0.45–1.84) or multiple logistic regression (OR 0.73, 95% CI: 0.39–1.37). On doing a multiple regression analysis with 1:1 matching of cases and controls (382 infection cases with 382 facility- and time-matched controls), cadre remained nonsignificant (OR 0.66, 95% CI: 0.30–1.46). Type of PPIUCD training was a significant covariate of infection even after adjusting for confounders; the odds of infection were lower for both onsite training (OR 0.45, 95% CI: 0.27–0.75) and on-the-job training (OR 0.31, 95% CI: 0.25–0.37) when compared with centralized training. Type of insertion and type of facility were not significant.

## 4 Discussion

As PPIUCD services are rolled out to the primary care level in the Indian public health system, it is important to understand whether the nurses and midwives who conduct most deliveries are able to provide PPIUCD services as safely and effectively as physicians. This case–control study demonstrates that two key negative outcomes — expulsion and infection — are not associated with the cadre of provider.

These results suggest that task sharing, that is, allowing nurses and midwives to take on tasks previously limited to physicians, is a safe and effective way to address the shortage of health workers. Shortage of health workers is a key constraint on access to FP services globally and to postpartum FP services, especially the PPIUCD, in India [24]. Allowing nurses to insert PPIUCDs also has the potential to increase acceptance of the method, as found by a study done in Turkey and in the Philippines [19]. Acceptance increased because nurses were more accessible and acceptable to clients than were physicians. A study in Zambia demonstrated the success of a program in expanding access to IUCD and implant services by competent midwives; after 14 dedicated midwives were made competent in IUCD insertions, acceptance of the IUCD at their busy clinics increased compared to other long-acting, reversible contraceptives [25]. IUCD prevalence has been shown to increase by more than two fold when nurses were allowed to perform insertions as a matter of policy in Turkey [26]. The study conducted in Turkey and in the Philippines also showed that client follow-up is improved when IUCD insertions are performed by nurses [19].

Competency-based training and posttraining support to enhance providers' proficiency are critical for providing good-quality PPIUCD services because the likelihood of expulsion depends on the technique of insertion. The fundal placement of the IUCD using correct technique reduces the chances of expulsion [27], [28]. Unexpectedly, our analysis found that the training approach was significantly associated with both expulsion and infection — but in opposite directions for the two outcomes. Expulsions were less likely, but infections were more likely, when providers had received centralized training rather than onsite or on-the-job training, even though the content of the training was standardized across all the training modalities. Poorer expulsion outcomes for providers who attended onsite and on-the-job training might be due to the inadequate number of clients available for supervised insertions at the peripheral health centers where these trainings are held. At tertiary facilities where centralized trainings are held, higher caseload ensures an adequate number of clients desiring PPIUCD insertions during training. In contrast, onsite and on-the-job training permits demonstration of infection prevention practices to providers in their own facility setting, catalyzing continuation of newly learned practices. We cannot draw conclusions about the effectiveness of different types of training in minimizing adverse outcomes in PPIUCD insertions because it was not the subject of this study. However, the findings suggest that further research is needed to explore this issue.

There are some limitations on the interpretation of the study findings. Although individual providers could have performed more than one insertion included in the dataset and this can shift the ORs in either direction, it was impossible to adjust for provider-level clustering of observations because the records did not include a provider identification number or standardized provider name. In addition, information on providers' experience with FP and PPIUCD insertion was frequently missing, and certain variables that are known to affect the likelihood of expulsion or infection, like preexisting medical conditions, were not recorded in the service registers.

In conclusion, the results of this study show that training nurses and midwives who conduct deliveries to insert IUCDs during the postpartum period has the potential to increase women's access to PPIUCD services at public health facilities without jeopardizing the quality of care.

## Figures and Tables

**Table 1:** Distribution of expulsion cases and controls, according to characteristics of facility, provider, insertion and follow-up

Characteristic	Expulsion cases (*n* = 792), frequency (%)	Controls (*n* = 1041), frequency (%)	p Value
**State**			
Bihar	396 (50.0)	458 (44.0)	**<.001**⁎
Chhattisgarh	50 (6.3)	60 (5.8)	
Jharkhand	27 (3.4)	29 (2.8)	
MP	228 (28.8)	239 (23.0)	
Rajasthan	09 (1.1)	14 (1.3)	
UK	40 (5.1)	44 (4.2)	
UP	42 (5.3)	197 (18.9)	
Missing	0	0	
**Type of health facility**			
Primary health center (PHC)/community health center (CHC)/subdistrict hospital (SDH)	162 (20.5)	161 (15.5)	**.003**⁎
District hospital	552 (69.7)	738 (70.9)	
Medical college	78 (9.8)	142 (13.6)	
Missing	0	0	
**Qualification of provider**			
Auxiliary nurse midwife	80 (10.1)	79 (7.6)	**<.001**⁎
General nurse midwife (GNM)	382 (48.2)	403 (38.7)	
BSc Nursing	51 (6.4)	52 (5.0)	
Medical graduate	53 (6.7)	88 (8.5)	
Postgraduation	140 (17.7)	306 (29.4)	
Missing	86 (10.9)	113 (10.9)	
**Experience of provider, in years (mean ± SD)**			
In offering FP	11.49 ± 9.14	13.23 ± 9.24	**<.001**⁎⁎
Missing	198	283	
In PPIUCD insertions	1.20 ± 0.97⁎	1.28 ± 0.77	.113⁎⁎
Missing	325	405	
**Type of training**			
Centralized	417 (52.7)	578 (55.5)	**<.001**⁎
Onsite	176 (22.2)	103 (9.9)	
On the job	185 (23.4)	209 (20.1)	
Missing	14 (1.8)	151 (14.5)	
**Type of insertion**			
Postplacental	464 (58.6)	570 (54.8)	**<.001**⁎
Postpartum	236 (29.8)	251 (24.1)	
Intracesarean	86 (10.9)	190 (18.3)	
Missing	6 (0.8)	30 (2.9)	
**Type of follow**-**up**			
Clinical visit	626 (79.0)	680 (65.3)	**<.001**⁎
Telephone	166 (21.0)	361 (34.7)	
Missing	0	0	

⁎ By chi-square test.

⁎⁎ By independent sample *t* test.

**Table 2:** Distribution of infection cases and controls, according to characteristics of facility, provider, insertion and follow-up

Characteristic	Infection cases (*n* = 382), frequency (%)	Controls (*n* = 1041), frequency (%)	p Value
**State**			
Bihar	199 (52.1)	458 (44.0)	**<.001**⁎
Chhattisgarh	10 (2.6)	60 (5.8)	
Jharkhand	01 (0.3)	29 (2.8)	
MP	30 (7.9)	239 (23.0)	
Rajasthan	05 (1.3)	14 (1.3)	
UK	25 (6.5)	44 (4.2)	
UP	112 (29.3)	197 (18.9)	
Missing	0	0	
**Type of health facility**			
PHC/CHC/SDH	65 (17.0)	161 (15.5)	.714⁎
District hospital	269 (70.4)	738 (70.9)	
Medical college	48 (12.6)	142 (13.6)	
Missing	0	0	
**Qualification of provider**			
ANM	36 (9.4)	79 (7.6)	**.011**⁎
GNM	147 (38.5)	403 (38.7)	
BSc Nursing	03 (0.8)	52 (5.0)	
Medical graduate	38 (9.9)	88 (8.5)	
Postgraduation	118 (30.9)	306 (29.4)	
Missing	40 (10.5)	113 (10.9)	
**Experience of provider in years (mean ± SD)**			
In FP	10.73 ± 8.23	13.23 ± 9.24	**<.001**⁎⁎
Missing	116	283	
In PPIUCD	1.47 ± 1.67⁎	1.28 ± 0.77	.087⁎⁎
Missing	131	405	
**Type of training**			
Centralized	298 (78.0)	578 (55.5)	**<.001**⁎
On site	24 (6.3)	103 (9.9)	
On the job	33 (8.6)	209 (20.1)	
Missing	27 (7.1)	151 (14.5)	
**Type of insertion**			
Postplacental	208 (54.5)	570 (54.8)	.006⁎
Postpartum	99 (25.9)	251 (24.1)	
Intracesarean	73 (19.1)	190 (18.3)	
Missing	2 (0.5)	30 (2.9)	
**Type of follow**-**up**			
Clinical visit	327 (85.6)	680 (65.3)	**<.001**⁎
Telephonic	54 (14.1)	361 (34.7)	
Missing	1 (0.3)	0	

⁎ By chi-square test.

⁎⁎ By independent sample *t* test.

**Table 3:** Simple and multiple logistic regression analysis of covariates of expulsion of PPIUCD by 6 weeks after insertion

Predictor variable	Unadjusted OR (95% CI)	Adjusted⁎ OR (95% CI)
**Cadre of provider**		
Doctor (reference group)	1.00	1.00
Nurse	2.06 (0.99–4.26)	1.84 (0.82–4.12)
**Type of insertion**		
Intracesarean (reference group)	1.00	1.00
Postplacental	**1.86 (1.01–3.43)**	1.05 (0.67–1.63)
Postpartum	**2.16 (1.12–4.17)**	1.39 (0.90–2.14)
**Type of training**		
Centralized (reference group)	1.00	1.00
Onsite	**2.31 (1.91–2.80)**	**2.32 (1.86–2.89)**
On the job	**1.20 (1.07–1.35)**	**1.23 (1.11–1.36)**
**Type of facility**		
Medical college (reference group)	1.00	1.00
PHC/CHC/SDH	1.94 (0.82–4.59)	1.28 (0.75–2.19)
District hospital	1.37 (0.77–2.43)	1.11 (0.63–1.96)
**Type of follow**-**up**		
Clinic	1.00	1.00
Telephonic	**0.52 (0.40–0.67)**	**0.38 (0.29–0.49)**

⁎ Adjusting for type of insertion, type of training, type of facility and type of follow-up.

**Table 4:** Simple and multiple logistic regression analysis of covariates of infection at 6 weeks after PPIUCD insertion

Predictor variable	Unadjusted OR (95% CI)	Adjusted⁎ OR (95% CI)
**Cadre of provider**		
Doctor (reference group)	1.00	1.00
Nurse	0.91 (0.45–1.84)	0.73 (0.39–1.37)
**Type of insertion**		
Intracesarean (reference group)	1.00	1.00
Postplacental	0.98 (0.58–1.67)	1.18 (0.77–1.80)
Postpartum	1.07 (0.92–1.23)	1.23 (0.94–1.62)
**Type of training**		
Centralized (reference group)	1.00	1.00
Onsite	**0.44 (0.27–0.71)**	**0.45 (0.27–0.75)**
On the job	**0.30 (0.24–0.37)**	**0.31 (0.25–0.37)**
**Type of facility**		
Medical college (reference group)	1.00	1.00
PHC/CHC/SDH	1.27 (0.32–4.97)	1.48 (0.55–3.93)
District hospital	1.08 (0.54–2.16)	1.28 (0.66–2.47)
**Type of follow**-**up**		
Clinic	1.00	1.00
Telephonic	**0.32 (0.24–0.43)**	**0.44 (0.32–0.61)**

⁎ Adjusting for type of insertion, type of training, type of facility and type of follow-up.
